# Robotic stereotactic ablative radiotherapy for renal cell carcinoma in patients with impaired renal function

**DOI:** 10.1186/s12894-019-0531-z

**Published:** 2019-10-21

**Authors:** C. Senger, A. Conti, A. Kluge, D. Pasemann, M. Kufeld, G. Acker, M. Lukas, A. Grün, G. Kalinauskaite, V. Budach, J. Waiser, C. Stromberger

**Affiliations:** 1 Department of Radiation Oncology, Charité - Universitätsmedizin Berlin, Augustenburger Platz 1, 13353 Berlin, Germany; 2 Charité CyberKnife Center, Charité - Universitätsmedizin Berlin, Augustenburger Platz 1, 13353 Berlin, Germany; 3 Department of Neurosurgery, University of Messina, Messina, Italy; 4 Department of Neurosurgery and Center for Stroke research Berlin (CSB), Charité - Universitätsmedizin Berlin, Charitéplatz 1, 10117 Berlin, Germany; 5 Berlin Institute of Health (BIH), Anna-Louisa-Karsch-Str. 2, 10178 Berlin, Germany; 6 Department of Nuclear Medicine, Charité - Universitätsmedizin Berlin, Charitéplatz 1, 10117 Berlin, Germany; 7 Department of Nephrology and Medical Intensive Care, Charité - Universitätsmedizin Berlin, Charitéplatz 1, 10117 Berlin, Germany

**Keywords:** CyberKnife, Radiosurgery, SABR, Renal cell carcinoma, Kidney, Motion tracking

## Abstract

**Background:**

Robotic stereotactic ablative radiotherapy (SABR) is currently under investigation as a noninvasive treatment option for patients with renal cell carcinoma (RCC). For radiation therapy of RCC, tumor motion and the need for high ablative doses while preserving the remaining renal parenchyma is a challenge. We aimed to analyze the safety and efficacy of robotic radiosurgery in RCC in a specific difficult subgroup of patients with impaired renal function.

**Methods:**

We retrospectively identified all patients with RCC, treated with robotic SABR and motion compensation in our institution between 2012 and 2017. Either single fraction SABR of 24 or 25 Gy or 3 fractions of 12 Gy prescribed to the 70% isodose line was applied. Local control, overall survival, radiation side effects were evaluated together with renal function and tumor motion.

**Results:**

We analyzed data of 13 lesions treated in 10 patients with clear cell RCC and a mean age of 70.5 ± 13.6 years (range: 48–87). Prior to SABR, 8 patients underwent previous complete and/or partial nephrectomy, 7 patients presented with chronic kidney disease ≥ stage 3. The median of minimum, mean and maximum planning target volume doses were 23.2, 29.5 and 35.0 Gy for single fraction and 24.4, 42.5 and 51.4 Gy for the three fractions regime. Persistent local control by robotic SABR was achieved in 9 out of 10 patients (92.3% of all lesions) within a median follow-up period of 27 month (range: 15–54). One patient underwent nephrectomy due to progressive disease and sufficient renal function of the contralateral kidney. Renal function remained stable with a mean estimated glomerular filtration rate (eGFR) of 51.3 ± 19.7 ml/min at baseline and 51.6 ± 25.8 ml/min at follow-up. The largest respiratory-induced tumor motion was seen in superior-inferior direction, compensated by the CyberKnife with mean targeting errors of maximal 2.2 mm.

**Conclusions:**

Robotic SABR is technically feasible for the treatment of RCC in preexisting kidney disease with good local tumor control at about 2 years follow-up. Robotic SABR with motion tracking offers a valid treatment option for patients, who are at increased risk for progression to end-stage renal disease due to partial nephrectomy or ablative techniques.

## Background

Renal cell carcinoma (RCC) is the most common form of kidney cancer and its incidence has risen in recent years [1]. Due to increased incidental detection rates of kidney tumors, more RCC are still confined to the kidney at the time of diagnosis. The standard treatment for Stage I RCC is a partial renal resection. Radical nephrectomy is only performed for centrally located tumors or when partial resection is not feasible. Patients with bilateral tumors, contralateral recurrent tumor after unilateral nephrectomy, metastases from RCC in the contralateral kidney or preexisting chronic kidney disease are special candidates for partial nephrectomy. In these patients preserving renal parenchyma is essential to avoid chronic kidney disease.

As a possible therapeutic approach robotic stereotactic ablative radiotherapy (SABR) is currently under investigation as a non-invasive treatment option for patients with RCC. Renal cell carcinoma is frequently reported as a radio-resistant tumor. However, pathologic complete responses have been described after ablative radiotherapy previously [2]. Tumor motion and the need for high ablative radiation doses while preserving the remaining renal parenchyma, poses a major challenge. Robotic radiosurgery allows continuous tumor tracking under free breathing and therefore minimal gross tumor volume (GTV) to planning target volume (PTV) margins are needed. Robotic SABR for moving tumors is already established as a standard treatment option for patients with early stage non-small cell lung cancer [3, 4].

Although current data seem to demonstrate that SABR provides good tumor control while preserving the renal function [5, 6], most studies are limited to patients with normal renal function. In this study, we analyzed the safety and efficacy of image-guided CyberKnife (Accuray Inc., Sunnivale, USA) radiosurgery in RCC in a specific subgroup of patients with preexisting impaired renal function. Feasibility and technical aspects of robotic SABR will be provided as well.

## Methods

### Study design

Retrospective analysis of patient data was approved by the Ethics Committee Campus Charité Mitte (EA1/233/18). We identified all histology proven RCC patients, who were treated with robotic SABR in our center between June 2012 and April 2017. We collected data on patient characteristics regarding disease stage, preexisting kidney disease, estimated glomerular filtration rate (eGFR), clinical outcome, complications, local tumor control and overall survival. Dose-volume parameters were analyzed including prescription dose, fractionation, treatment dose (D_min_, D_mean_, D_max_,), GTV, PTV, new conformity index (nCI), PTV coverage, tumor motion and tracking accuracy.

### Robotic SABR planning and delivery

The patients were referred to CyberKnife irradiation from the nephrology department, all at increased risk for progression to end-stage renal disease caused by further invasive treatment. The decision to perform a robotic SABR was recommended by a multidisciplinary urology board review for patients who are at increased risk for progression to end-stage renal disease due to partial nephrectomy or other ablative techniques.

One gold fiducial marker (1.0 mm × 5.0 mm) was implanted within or close to each tumor using an 18-G needle under computed tomography (CT)-guidance in local anesthesia. A tissue sample was taken in the same procedure if there was no prior pathology report available. High-resolution native thin-slice (1.0 mm) planning CT was performed within a median of 8 days (range: 1–21) after fiducial insertion to allow for fiducial settlement [7]. For accurate tumor delineation, magnetic resonance images (MRI) were co-registered with the planning CT and contouring was performed on all axial slices. The GTV was defined as the tumor volume based on CT and MR images. The clinical target volume (CTV) was equivalent to the GTV. The PTV was obtained by adding in median a 3 mm (range: 0–5 mm) isotropic margin to the GTV. Depending on tumor size or organs at risk (OAR) two different dose concepts were used, either single fraction SABR of 24 or 25 Gy, or 36 Gy in 3 fractions (12 Gy/fraction) prescribed each to the 70% isodose covering the PTV. Treatment planning and dose calculations were obtained by MultiPlan 4.6 (Accuray Inc., Sunnyvale, USA) using the Ray-tracing algorithm.

The linear-quadratic model, assuming an a/ß ratio of 2.6–6.9 Gy for RCC [8], was used to calculate the biologically equivalent dose (BED) and the equivalent dose in 2 Gy fractions (EQD2). The calculated BED_6.9_ and EQD2_6.9_ encompassing the PTV for single fraction were 107.5 Gy and 83.3 Gy, and 98.6 Gy and 76.4 Gy for the 3-fraction treatment.

Dose constraints for OAR for single fraction treatments were as follows: < 5 cm^3^ of small bowel loops could receive up to 10.0 Gy with a maximum point dose of 19.0 Gy; for the extratumoral kidney parenchyma < 200 cm^3^ could receive up to 8.0 Gy. The normal tissue constraints for three fractions were: < 5 cm^3^ could receive up to 16.0 Gy with a maximum point dose of 27.0 Gy for small bowel, and less than 33% of the remaining kidney parenchyma could receive a total of 15.0 Gy. The dose constraints for spinal cord, liver, stomach and large intestine were set according to published standard limits [9]. The nCI [(*V*70% ∙ *V*_*PTV*_)/*V*70%_*PTV*_^2^], which describes the conformity between the prescription isodose and the volume and shape of the PTV, was also used for treatment plan evaluation.

### Technical aspects

The CyberKnife System installed in July 2011 in Berlin combines two systems, a lightweight linear accelerator mounted on a robotic arm with 6-MV photon energy and an image guidance system consisting of two orthogonally positioned x-ray cameras. For patient positioning, an automatic tracking algorithm compares live x-rays with digital reconstructed images from planning CT. For respiratory motion compensation, the CyberKnife Synchrony® Respiratory Motion Tracking System (MTS) was used. Thereby, the external motion of LED markers located on the chest of the patient was correlated with the internal tumor motion represented by the fiducial position and determined by the x-ray images. The individually measured correlation model is continuously updated and synchronizes the radiation beam in real time such that the beam always remains aligned with the target. An accuracy of less than 1.0 mm is technically achieved and allows clinicians to reduce safety margins significantly, while eliminating the need for gating or breath-hold techniques. During treatment, the motion patterns for each patient were recorded in logfiles.

### Follow-up and statistics

Clinical and radiological follow-up with CT or MRI was frequently performed after robotic SABR and the latest available follow-up was used in this analysis. For local control the MRI scans were evaluated by the senior physician in charge to verify treatment response. Tumor response was analyzed using response evaluation criteria in solid tumors (RECIST version 1.1). The treatment response of each RCC was categorized using OsiriX MD 10.0 (Pixmeo SARL, Bernex, Switzerland) to compare baseline MRI and planning CT with the latest available follow up images in 1) complete remission (CR): no measurable lesion; 2) partial remission (PR) defined as a volume reduction ≥ 30%; 3) stable disease (SD); 4) progressive disease (PD) defined as a *≥* 20% increase in volume or *≥* 5 mm increase in size. Local control (LC) was calculated from the end of SABR until last available follow-up or PD.

Overall survival (OS) was calculated from the end of SABR until last follow-up or death. LC and OS were estimated using Kaplan-Meier curves. Common Terminology Criteria for Adverse Event V4.03 (CTCAE) for acute and late radiosurgery related side effects were recorded separately. Renal function at baseline and latest available follow up was calculated according to the chronic kidney disease epidemiology collaboration (CKD-EPI) formula at baseline and last available follow up.

Due to respiratory induced kidney motion, the motion patterns and total targeting errors between the predicted and the actual position of the tumor were evaluated. Overall, the motion pattern and targeting accuracy of 19 out of 21 treatment sessions could be extracted. Motion pattern evaluation and statistical analysis were done with MATLAB 9.3 (The MathWorks, Inc., Natick, USA).

## Results

### Patient characteristics

Data of 13 lesions treated in 10 patients histologically confirmed as clear cell RCC grade 1 or 2 were collected. The mean age of patients who underwent robotic SABR was 70.5 ± 13.6 years (range: 48–87). The female/male ratio was 1:1. All patients treated with robotic SABR had an ECOG performance status 1 or 2 and suffered from chronic kidney disease (CKD). The median and mean time interval between the first histological diagnosis of RCC and SABR was 7.5 and 8.4 ± 6.0 years, respectively, with a large range of 2 months to 19.7 years. Tumor characteristics are summarized in Table 1. Seven patients had T1a (≤ 40 mm) and 3 patients had larger tumors (T3a). In 3 patients it remained unclear whether the treated tumor was a metachronous RCC or a metastasis from a previously occurred contralateral RCC. The subsites of the 13 lesions were the upper, mid or lower pole in 53.8%, close to the renal pelvis or extending to the perinephric tissue in 15.4% each and infiltrating the renal vein or close to the hilum in 7.7% each.

**Table 1:** Tumor characteristics and preexisting kidney disease in patients with renal cell carcinoma

Case	Size (mm)	Primary tumor	Tumor location	Baseline CKD stage	First line treatment / Preexisting kidney disease
#1	32	cT1a/DD metastasis	close to renal pelvis	3b	Nephrectomy, RFA and embolisation ipsilateral
#2	30	cT1a/DD metastasis	mid pole	2	Nephrectomy, partial resection ipsilateral /DM type 2
#3	14 10	cT1a	upper pole mid pole	3b	Nephrectomy, partial resection ipsilateral / short term dialysis, DM type 2
#4	26	cT1a	upper pole	2	RPGN, kidney transplant
#5	70	cT3a	infiltrating renal vein	2	Partial resection contralateral
#6	36	cT1a	mid pole	4	Nephrectomy
#7	36	cT1a	lower pole	3a	partial resection contralateral, multiple RFA ipsilateral / VHL
#8	39	cT1a	close to renal pelvis	3a	partial resection ipsi- and contralateral / VHL
#9	47	cT3a	extends to perinephric tissue	3b	–
#10	9 10 15	cT3a/DD metastasis	lower pole extends to perinephric tissue close to hilum	3b	Nephrectomy, partial resection ipsilateral

*RFA* Radiofrequency ablation, *CKD* chronic kidney disease, *DM* diabetes mellitus, *RPGN* rapidly progressive glomerulonephritis, *VHL* von Hippel-Lindau disease

Prior to SABR, 8 out of 10 patients underwent surgery or radiofrequency ablation (RFA) for their renal tumors, 6 of them had procedures done on both sides. Nephrectomy was carried out in 5 patients, partial ipsilateral resection in 4 patients and contralateral resection in 3 patients. Previous RFA of the SABR treated kidney was performed in 2 patients. Von Hippel-Lindau disease was diagnosed in 2 patients. One patient had a RCC in his kidney transplant. Three patients had a diabetes mellitus type 2. CKD stage 2, 3 and 4 with an eGFR level below 90, 60, 45 ml/min was diagnosed in 3, 6 and one patient, respectively (see Table 1 for preexisting kidney disease).

### Treatment and Dosimetric analysis

The tumors had a median diameter of 28.8 mm (range: 9–70). Two patients had tumors larger than 40 mm with RCC extension into the renal vein or perinephric tissue. The median GTV volume was 13.3 cm^3^ (range: 1.3–108.4), the resulting median PTV was 22.1 cm^3^ (range: 3.8–190.3). Five patients received single fraction SABR of 24 or 25 Gy, 4 patients received 3 fractions of 12 Gy every other day and one patient with three lesions received both regiments. The patient’s median of minimum, mean and maximum PTV dose was 23.2, 29.5 and 35.0 Gy for single fraction and 24.4, 42.5 and 51.4 Gy for the three fractions regimen, respectively. Dose-volume parameters and further treatment characteristics including nCI and percentage of the PTV coverage are summarized in Table 2.

**Table 2:** Dose-volume and follow-up parameters for robotic stereotactic ablative body radiotherapy

Case	GTV (cm^3^)	PTV (cm^3^)	Margin (mm)	Dose (Gy)	PTV coverage (%)	nCI	Follow-up (month)	Local control
#1	8.2	17.9	4	1 × 24	97.8	1.07	54	SD
#2	20.4	31.0	3	1 × 25	97.8	1.06	23	PR
#3	13.5 17.0	13.5 17.0	0 0	1 × 24 1 × 24	96.8 98.5	1.13 1.13	47	CR PR
#4	14.3	24.6	3	1 × 25	99.9	1.06	33	SD
#5	108.4	190.3	5	3 × 12	92.0	1.23	25	SD
#6	13.2	22.7	3	1 × 25	99.5	1.13	15	SD
#7	9.2	17.4	3	3 × 12	98.3	1.14	32	SD
#8	45.5	88.4	5	3 × 12	86.0	1.40	30	PD
#9	44.2	66.2	3	3 × 12	98.7	1.21	23	PR
#10	1.3 9.0 2.3	3.8 21.4 5.6	3 4 3	1 × 25 3 × 12 1 × 25	99.7 82.0 98.4	1.09 1.50 1.20	16	CR PR CR

*GTV* Gross tumor volume, *PTV* planning target volume, *nCI* new conformity index, *SD* stable disease, *PR* partial remission, *CR* complete remission, *PD* progressive disease

Each robotic SABR treatment was done as an outpatient procedure with delivery times between 46 min and 86 min per session. For single session treatments, the mean total treatment time was 62 ± 15 min, fractionated treatments took in total 184 ± 33 min (61 ± 11 min per fraction). All patients completed their treatment.

### Tumor response

**Fig. 1:**
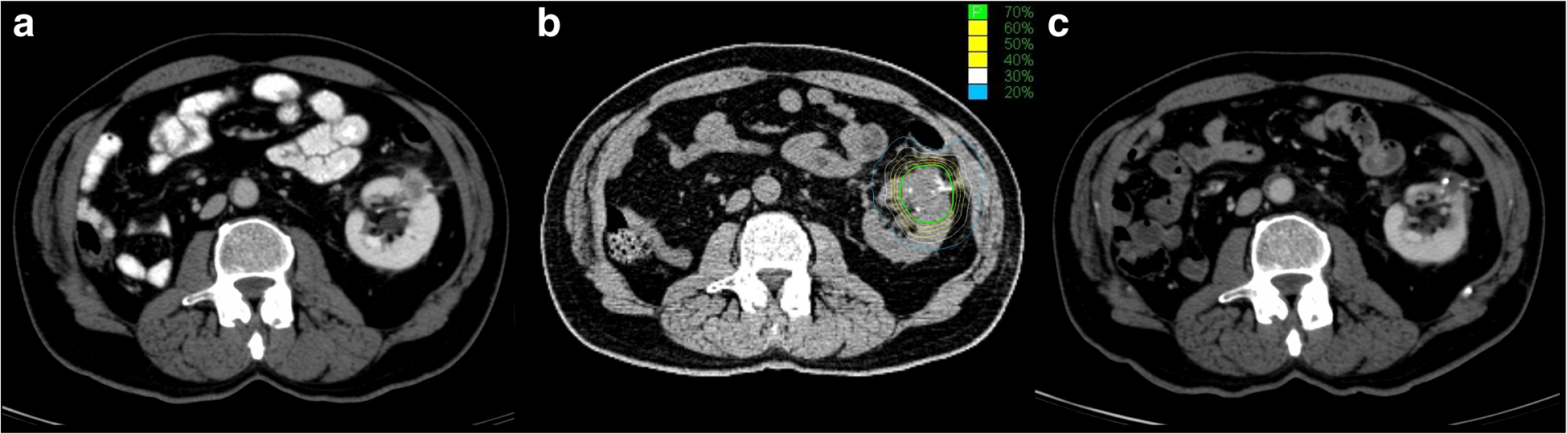
A representative case of a renal cell carcinoma. **a** Demonstrates a representative image before radiotherapy and **b** the treatment plan with exemplary planning computed tomography image in axial view with the treatment plan. Single fraction 25 Gy was prescribed to 70% isodose line (in green) to treat planning target volume shown in red line. The yellow and white circle lines represent the remaining isodose lines until 20% in blue. **c** shows an image 2 years after robotic stereotactic ablative radiotherapy

Local control (CR, PR and SD) by robotic SABR therapy was achieved in 9 out of 10 patients and 92.3% of all lesions within the median follow-up period of 27 month (range: 15–54). A representative example of the tumor response and treatment plan is shown in Fig. 1. Whereas SD was observed in 38.5% of the treated lesions, PR was observed in 30.8% and CR in 23.1% (Table 2). However, there was no difference in SD or PR between the one fraction or three fractions regiment. The only local treatment failure occurred in one lesion (7.7%) 5 month after SABR.

**Fig. 2:**
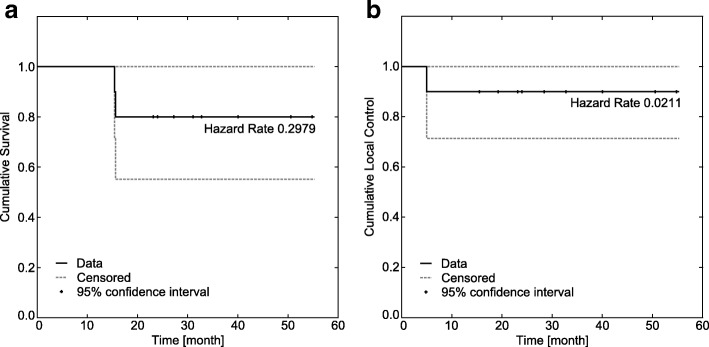
Kaplan-Meier curves. **a** Shows overall survival and **b** local control for renal cell carcinoma patients after robotic stereotactic ablative radiotherapy

Four patients with metastases to other organs at time of radiosurgery or during follow-up had additional adjuvant systemic treatment. Out of 10 patients 8 were alive at the last available follow-up. Two patients with progressive metastatic disease died 15 and 16 months after SABR. Kaplan-Meier Curves for local control and overall survival are shown in Fig. 2.

### Renal function and toxicity

**Fig. 3:**
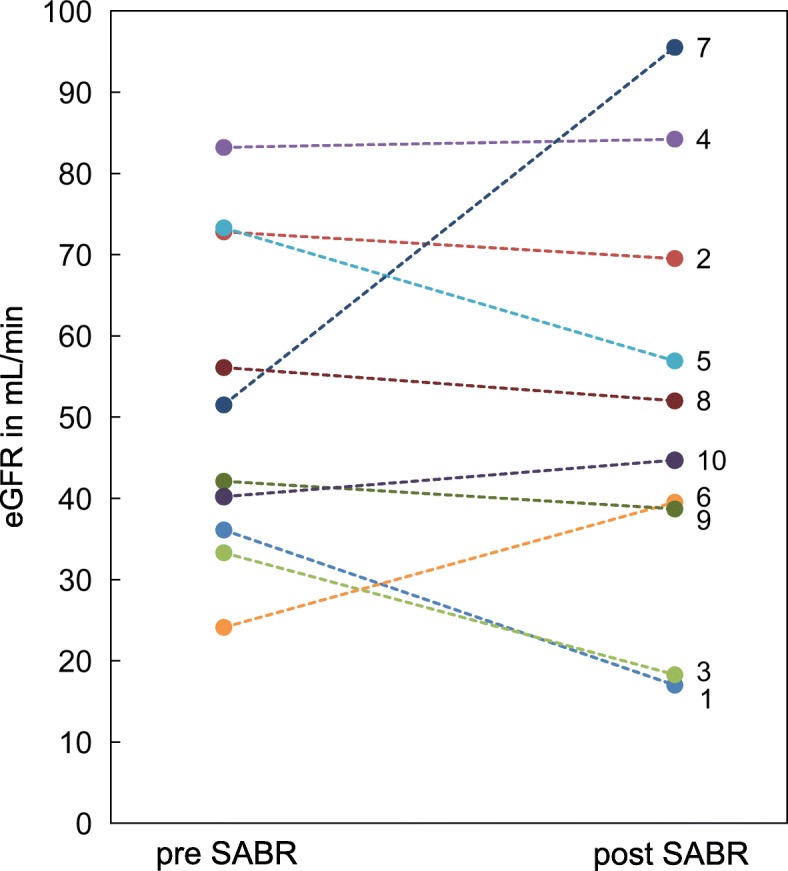
Kidney function at baseline and follow-up. Estimated glomerular filtration rates (eGFR) before stereotactic ablative radiotherapy (pre SABR) and latest available follow-up (post SABR)

Typical normal tissue dose constraints were within the range mentioned. Over a median follow-up period of 22 month (range: 4–51) renal function remained stable with a mean serum creatinine of 1.4 ± 0.5 mg/dl (eGFR 51.3 ± 19.7 ml/min) at baseline and 1.5 ± 0.8 mg/dl (eGFR 51.6 ± 25.8 ml/min) at follow-up (Fig. 3). One patient underwent nephrectomy due to progressive disease after SABR with three fractions of 12 Gy and sufficient renal function of the contralateral kidney. One patient developed mild abdominal pain (grade 1) and another one diarrhea and abdominal distension (grade 1). All symptoms occurred in the two patients with tumors larger than 40 mm. No patient developed CTCAE grade 2 or higher toxicity or needed hemodialysis.

### Tumor motion tracking

**Fig. 4:**
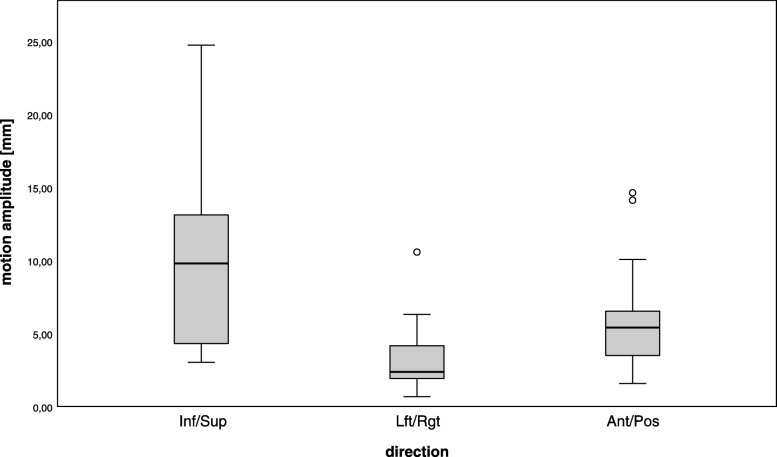
A diagram of motion amplitudes. Maximal motion amplitudes of all treatment sessions in inferior/superior (Inf/Sup), left/right (Lft/Rgt) and anterior/posterior (Ant/Pos) direction in mm

Each patient got one gold fiducial implanted per lesion. Two patients had 2 and 3 gold fiducials for multiple lesions. There were no side effects with marker placement in the kidney or difficulties with marker migration observed. To position the patient as in planning CT, he was first aligned using the bony spine structures. Afterwards the position of the fiducial was tracked. The majority of the lesions (92.3%) were treated using MTS for motion compensation. The only robotic SABR done without tumor motion tracking was performed in a kidney transplant located in the left iliac fossa where no respiratory motion was suspected. For all patients, treatment was performed in “free-breathing”, the largest respiratory-induced tumor motion was seen in superior-inferior direction with magnitudes between 3.0 mm and 24.7 mm. The left/right and anterior/posterior displacements of the tumor ranged from 0.7 to 10.6 mm, and 1.6 to 14.6 mm, respectively (Fig. 4). This motion was compensated by the CyberKnife with mean targeting errors over the complete treatment time of maximal 2.2 mm.

## Discussion

In this retrospective study the efficacy of robotic SABR was assessed retrospectively in 10 patients with RCC and moderate to severe chronic kidney disease. Our study demonstrates that this minimal invasive and highly sophisticated treatment method provides good response rates and local control with negligible toxicity. SABR with motion compensation is a nephron-sparing treatment that perfectly adapts to patients with RCC and significant preexisting chronic renal failure.

Our results concerning local tumor control in 92.3% of all lesions and mild toxicity appear to be consistent with those available in the literature. A previous systematic review of 126 patients described a weighted local control rate of 94% and a grade 3 toxicity rate of 3.8% [10]. Since that study, 3 single-institution, prospective studies of 19 patients [11], 40 patients [2], and 33 patients [12] have reported similar findings, with local control rates ranging from 98 to 100% and grade > 3 toxicity rates from 0 to 15.8%. Recently, 9 centers across Germany, Australia, the United States, Canada, and Japan formed an International Radiosurgery Oncology Consortium for Kidney and reported data of 223 patients [6]. The rates of LC, cancer-specific survival, and progression-free survival at 2 and 4 years were 97.8, 95.7, 77.4% and 97.8, 91.9, 65.4%, respectively. Multi-fraction SABR was associated with poorer progression-free survival and worse cancer-specific survival. Grade 1 and 2 toxicities were reported for 35.6% of patients whereas grade 3 and 4 toxicities were recorded in only 1.3% [6].

In patients with bilateral tumors or contralateral tumor recurrences after unilateral nephrectomy or partial resection treatment is especially challenging. The resection of the remaining kidney consecutively leads to the progression of chronic kidney disease including the need for hemodialysis treatment. In such cases, minimally invasive ablative techniques such as cryosurgery, radiofrequency ablation and SABR are possible alternatives to nephrectomy. A 2016 systematic review and meta-analysis reporting on survival across management strategies demonstrated a 95 to 100% cancer specific survival after nephrectomy and thermal ablation with a median follow-up period of 22 to 120 months. Whereas, for tumors more than 40 mm (T1b) survival rates decrease to around 90% and for tumors more than 70 mm (T2) between 82.5 and 86.7% [13]. A mostly retrospective data analysis by Kunkle and Uzzo [14] showed local tumor progression rates of 12.9% after RFA and 5.2% after renal cryoablation. In our series, tumor progression was recorded in 7.7% of all lesions, accordingly in one out of 10 patients (10%). In this case the tumor size was above the median, located close to renal pelvis and the PTV coverage was less than 90%.

Notably, renal function remained stable following treatment in all patients despite the high doses of radiation delivered to the kidney. This result raises two considerations. Firstly, preservation of renal function was assumed to be due to compensatory mechanisms of the contralateral kidney and the spared ipsilateral kidney volume described as renal functional reserve [15]. These results also suggest that it might be possible to rely on a compensatory capacity of the ipsilateral kidney in patients who already had contralateral nephrectomy and that, whenever oncologically suitable, a selective approach aimed to avoid post-treatment severe chronic kidney disease should be pursued. A second point concerns the radiation tolerance of the peritumoral kidney and the reliability of tumor tracking in robotic SABR. Cassady [16] proposed a threshold dose of 15 Gy for renal injury based on data of bilateral whole kidney irradiation in 3 fractions. Nevertheless, ours and other previous studies demonstrate a good tolerance to higher doses and stable kidney function. The prescribed dose (1 × 24–25 Gy or 3 × 12 Gy prescribed to the 70% isodose) was relatively high in order to overcome the radio-resistance of RCC. The fraction number and prescribed dose of the two large studies from Staehler et al. [2] and Sun et al. [17] were similar to our dose concepts. Overall, they treated 80 patients with either 25 Gy in one fraction or 38 Gy in 3 fractions prescribed to the 70 or 80% isodose line. Both studies reported only grade 1 side effects with > 90% local control in a relatively short follow-up.

Svedman et al. [18] evaluated kidney injury following 3 fractions SABR in 7 patients with primary or metastatic renal disease with only one functioning kidney. In 5 patients, kidney function remained unaffected after SABR, with a kidney volume of 37.3% receiving 15 Gy (V15), whereas 2 patients exhibited modest changes in renal function without the requirement for medical intervention or hemodialysis. In SABR patients, a V15 limited to less than one third of the normal single remaining kidney could be an appropriate dose-volume constraint in patients with preexisting kidney disease. We therefore considered this dose constraint in our series for the three-fraction regiment.

Furthermore, the high doses used and the treatment result in terms of remission, local control and sparing of renal function, demonstrate that the robotic SABR is highly reliable in terms of targeting precision and dose delivery. According to our data, the median targeting accuracy was within 2.2 mm. This provided us an important information regarding the margins to be used. In fact, we believe that, unlike margins of up to 10 mm, as used in other studies, a moderate expansion of the tumor (i.e. 3.0 mm) is sufficient for the CyberKnife MTS. Since only one marker was implanted, rotations could neither be directly detected nor corrected. However, geometric calculations have shown that a 3.0 mm margin appears to be sufficient also if small rotations (< 5 °) occur.

### Limitations

This study has several limitations. This is a retrospective series with a limited number of cases collected and a relatively short follow-up for renal function. Nevertheless, it should be considered as a proof-of-concept study for SABR on patients with impaired renal function gaining satisfactory results and providing a low risk for treatment-related side effects.

## Conclusion

Robotic SABR is technically feasible for the treatment of early stage RCC in patients with preexisting kidney disease with good local control at short term follow-up. As an outpatient procedure, it may prevent (treatment related) loss of renal function with only mild side effects. Therefore robotic SABR with motion tracking represents a valid treatment option for these patients, who are at increased risk for progression to end-stage renal disease due to partial nephrectomy or other ablative techniques. Further studies are needed but warranted to determine long-term results of this treatment.

## Data Availability

Statistical data from the present study is available from the corresponding author on reasonable request.

## Abbreviations

CR: Complete remission
CT: Computed tomography
CTV: Clinical target volume
eGFR: Estimated glomerular filtration rate
GTV: Gross tumor volume
LC: Local control
MRI: Magnetic resonance images
MTS: Motion Tracking System
nCI: New conformity index
OAR: Organs at risk
OS: Overall survival
PD: Progressive disease
PR: Partial remission
PTV: Planning target volume
RCC: Renal cell carcinoma
RFA: Radiofrequency ablation
SABR: Stereotactic ablative radiotherapy
SD: Stable disease
